# Effects of Radial Extracorporeal Shock Wave Therapy on Flexor Spasticity of the Upper Limb in Post-stroke Patients: Study Protocol for a Randomized Controlled Trial

**DOI:** 10.3389/fneur.2021.712512

**Published:** 2021-09-09

**Authors:** Tao Fan, Xiangying Zhou, Peichen He, Xiaojia Zhan, Peng Zheng, Rong Chen, Rongdong Li, Rihui Li, Mingyang Wei, Xue Zhang, Guozhi Huang

**Affiliations:** ^1^Department of Rehabilitation Medicine, Zhujiang Hospital, Southern Medical University, Guangzhou, China; ^2^School of Rehabilitation Medicine, Southern Medical University, Guangzhou, China

**Keywords:** extracorporeal shock wave therapy, stroke, hemiplegia, spasticity, neurorehabilitation

## Abstract

**Background:** Flexor spasticity of the upper limb is common in poststroke patients and seriously affects the recovery of upper limb function. However, there are no standard management protocols for this condition. Radial extracorporeal shock wave therapy (rESWT) is widely used for various diseases, some studies reported the effects of ESWT on reducing spasticity, but the mechanism of ESWT to reduce spasticity by affecting the excitability of stretch reflex or non-neural rheological components in spastic muscles or both is not yet clear. A large randomized controlled trial with comprehensive evaluation indicators is still needed. The study is to observe the effect of rESWT on flexor spasticity of the upper limb after stroke and explore its mechanism.

**Methods:** A prospective, randomized, double-blind controlled trial is to be performed. One hundred participants will be recruited from the Inpatient Department of Zhujiang Hospital. Eligible patients will be randomly allocated to either receive three sessions of active rESWT (group A) or sham-placebo rESWT (group B) with 3-day intervals between each session. Assessment will be performed at baseline and at 24 h after each rESWT (t1, t2, and t3). The primary assessment outcome will be the Modified Ashworth Scale, and other assessments include surface electromyography, MyotonPRO digital muscle function evaluation, and infrared thermal imaging. All data will be analyzed using intention-to-treat principles. Multiple imputation by chained equations will be used to address missing data caused by loss to follow-up and nonresponses. Per protocol, analyses will also be performed on the participants who complete other assessments. Statistical analysis will be performed using SPSS software (version 20.0) and the significance level set at *p* < 0.05.

**Discussion:** This trial aims to analyze the application of rESWT for the management of spasticity after stroke *via* appropriate assessments. We hypothesized that after receiving active rESWT, patients would show greater improvement of upper limb muscles compared with patients within the sham-placebo group. The rESWT would be an alternative to traditional methods, and the results of this study may provide support for the further study of potential mechanisms.

**Clinical Trial Registration:**www.chictr.org.cn, identifier: ChiCTR1800016144.

## Background

Spasticity is a form of muscle hypertonia that results in the pyramidal tract when corticoreticulospinal fibers (extrapyramidal tract) are damaged, which gives an upper motor neuron syndrome due to a lesion of the central nervous system (brain and/or spinal cord) (1). After central nervous system injury, the inhibition of cerebral cortex and other higher centers is lost, and the excitability of γ-motor neurons is enhanced so that the excitability and sensitivity of muscle spindle are increased. Through the α-γ loop, the α-motor neurons in the anterior horn of the spinal cord are overexcited, resulting in an excessive increase in the stretch reflex. Continuous contractions of spasmodic muscles can lead to contracture, pain, limited joint movement, and joint deformity (2). Flexor spasticity is common in poststroke patients with upper limb dysfunction, and uncontrolled spasticity often leads to secondary complications such as pain and contractures (3) while placing a significant mental and financial burden on the patient, caregivers, and society (4). The mechanisms underlying this disorder may be due to the hyperexcitable stretch reflexes caused by the imbalance of supraspinal inhibitory and excitatory inputs after the upper motor neuron lesion (1) and also involve the changes in muscle properties (5, 6) (e.g., stiffness, fibrosis, and atrophy), which is referred as intrinsic hypertonia. Aggressive and appropriate spasticity management contributes to motor relearning and function recovery during chronic stages (4, 7). Currently, mainstream interventions for upper limb hypertonia include stretching, oral anti-spasticity medications, focal botulinum toxin (BTX) injections, and surgical treatment (7–10). However, the treatment methods mentioned earlier have advantages or drawbacks in their effectiveness and safety (8, 11–13). For example, BTX injection therapy is more effective but invasive and more expensive. Surgical treatment is more traumatic, and the risk is higher.

An extracorporeal shock wave is defined as a sequence of single sonic pulses with high peak pressure (14, 15), which can cause energy gradient differences and torsional tension between tissues of different densities through energy conversion and transmission, and form a cavitation effect, which induces a biological effect (16). During the past few decades, extracorporeal shock wave lithotripsy (17) has been widely used and has evolved to be standard therapy used for urinary calculi due to its excellent efficacy, non-invasiveness, and lack of obvious complications. At present, extracorporeal shock wave therapy (ESWT) has also been widely used for the treatment of various neurological and musculoskeletal diseases (18, 19), such as cerebral palsy (20), multiple sclerosis (21), tendinopathy (22), chronic tennis elbow (23), and nonunion of long bone fracture (24). Recently, several studies (25–27) have indicated that ESWT could be used for decreasing hypertonia in strokes. However, only a few convincing studies have been published; a systematic review (28) in 2020 evaluated the scientific reliability and methodological quality of recent clinical trials according to their level of evidence and found that among enrolled 17 studies, only seven studies obtained Sackett's grading system's highest level 1 of evidence (studies scoring 6–10). A most recent overview (29) in 2021 concluded that an ESWT effectively reduced spasticity after stroke without adverse effects, but the mechanism of action of ESWT on spasticity muscles and standard parameters of ESWT (regarding frequency, energy flux density, location, and total ESWT sessions) in poststroke spasticity remained unclear. Therefore, a prospective large-sample randomized, double-blind controlled trial with a comprehensive assessment method is needed to further confirm its efficacy and explore the underlying mechanism of ESWT on spasticity. There are two types of ESWT: focused extracorporeal shock wave therapy (fESWT) and radial extracorporeal shock wave therapy (rESWT). According to differences of mechanical properties in fESWT and rESWT and combine the study outcomes of Wu et al. (30), which was the only one to directly compare the effect of rESWT and fESWT so far, we chose rESWT device for the management of upper limb spasticity after stroke in this study.

The Modified Ashworth Scale (MAS) (31, 32) is the most widely used clinical scale to grade spasticity and shows high inter- and intra-rater reliability for the assessment of muscle tone of upper extremities. H reflex can objectively evaluate changes in the excitability of α-motor neurons before and after treatment. Motor nerve conduction velocity (MCV) is a diagnostic technique used to assess peripheral nerve conduction function. By detecting the MCV of the median nerve before and after rESWT intervention, we can observe whether the peripheral nerve has been damaged and determine the safety of the ESWT procedure. Surface electromyography (sEMG) can reflect the overall situation of muscle activity by placing surface electrodes on the muscle to collect electrical signals (33). The sEMG indexes of root mean square (RMS), integrated electromyogram (iEMG), and co-contraction ratio (CR) (34–36) can be used to assess muscle spasms objectively and quantitatively. The MyotonPRO digital muscle function assessment system is a new non-invasive instrument that can be used to evaluate the functional status of skeletal muscles and biological soft tissue. It can quantitatively measure muscle tension, elasticity, stiffness, and other functional conditions (37, 38). A previous study by Dymarek et al. (39) has shown that ESWT can improve trophic conditions of spastic muscles. The infrared thermal imaging (IRT) system detects local trophic condition changes in spasmodic muscles of the upper limbs, which are associated with blood microcirculation and surface temperature distribution (40, 41). The assessment techniques mentioned earlier can reflect the changes of upper limb spasm, muscle tension, nerve excitability, muscle elasticity, and surface temperature of stroke patients from different perspectives and can comprehensively reflect the effects of ESWT from multiple perspectives. Therefore, this trial is designed to use the assessment techniques mentioned earlier to examine the efficacy (rESWT can relieve upper limb spasticity after stroke) and safety (rESWT has no harm to nerve, muscle, and other tissues and organs) of rESWT for the treatment of upper limb spasticity after stroke.

## Methods/Design

### Trial Design

**Figure 1:**
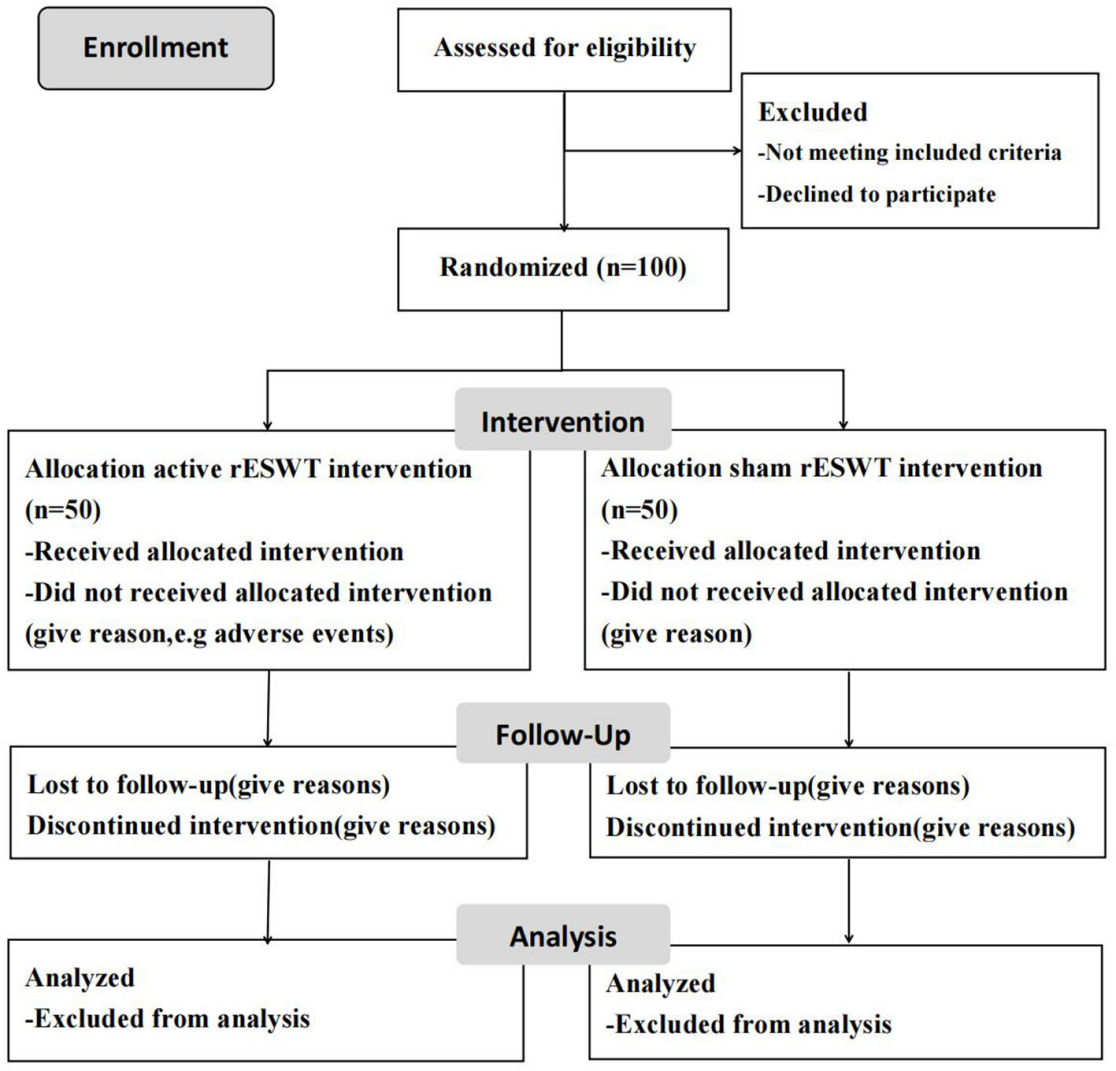
CONSORT flow diagram of study.

This study is a prospective, double-blind, and randomized controlled trial that conformed to Standard Protocol Items of the Recommendations for Interventional Trials guidelines (42) (Additional file 1). The flow diagram of the study to be followed in this study is shown in Figure 1. The participants are observed during their hospital stay and are randomly assigned to either receive three rounds of treatment with active rESWT (group A) or sham-placebo rESWT (group B). Assessments including MAS, H reflex, MCV, sEMG, myotonometer, and IRT are to be performed at baseline and at 24 h after intervention.

### Study Setting

The study setting is the Department of Rehabilitation Medicine at the Zhujiang Hospital of Southern Medical University, Guangzhou, China. The first patient was recruited on October 18, 2018, and the recruitment was planned to be completed within 3 years.

### Patient and Public Involvement

To have scientifically rigorous clinical trials and reliable results, patients in this trial will not be involved in the design, recruitment, or conduction of the study. After the last visit, the patients will be informed by the study's physician of the results in the form of pictures and text.

### Inclusion Criteria

Participants will be qualified for inclusion when they meet the following criteria: (1) meeting the “criteria for the diagnosis of cerebrovascular diseases” adopted by the Fourth Academic Conference on Cerebrovascular Diseases in 1995 (43) and confirmed using computed tomography or magnetic resonance imaging examination of the head; (2) age between 35 and 75 years old, with a first episode > 1 month and being in a stable clinical condition; (3) the presence of hemiplegic or hemiparesis with elbow joint spasticity grade > 1, determined by MAS (31); (4) no obvious cognitive impairment; and (5) the informed consent form being completed by the patient.

### Exclusion Criteria

The exclusion criteria are as follows: (1) having received oral antispasmodic drugs, BTX injections, and local block or surgical treatments for decreasing spasticity; (2) fixed elbow joint muscle contracture; (3) uncontrolled hypertension; (4) patients with chronic heart failure, malignant arrhythmia, and other severe organic heart diseases; (5) patients with a pacemaker and other electronic implants; (6) the presence of coagulation dysfunction; (7) patients with local infections and skin rupture; and (8) refusal to participate in this study.

### Interventions

**Active Radial Extracorporeal Shock Wave Therapy Intervention (Group A):** An rESW device (Power Shocker LGT-2510A, Guangzhou Longest Science & Technology Co., Ltd., China) was used for shock wave therapy. Study participants were treated in the supine position on the area of the biceps brachii, the flexor carpi radialis, and the pronator teres muscle after their affected side has been marked and the skin has been cleaned by soaking with alcohol.

The coupling gel as a contact medium is evenly applied onto the treatment probe to reduce tissue resistance, and a medium- to high-intensity pressure was applied to keep the probe close to each muscle belly. The following protocol is to be used: 2,000 shots, an energy flux density of 0.03 mJ/mm^2^, a pressure of 0.2 MPa (two bars), and a frequency of 8 Hz is delivered to each marked position, for a total of three rounds of treatment, once in 3 days.

**Sham Radial Extracorporeal Shock Wave Therapy Intervention (Group B):** The protocol to be used is the same as that of “group A,” except that pressure is one bar and no coupling gel is used on the treatment site, whereas a thick layer of gauze is placed between the skin and the probe instead, with no pressure applied.

The physiotherapist who is to perform the intervention should have received standardized training and must be familiar with the process and details of the intervention. The patients are all required to undergo the same routine therapeutic program (including common physical therapy and occupational therapy).

### Outcome Measures

#### Primary Outcome Measure: Modified Ashworth Scale

The scale is graded in six stages, ranging from 0 (no increase in tone) to 4 (limb rigid in flexion or extension). For the convenience of statistical analysis, a MAS grade of 1+ is to be substituted with a value of 2, whereas grades 2, 3, and 4 are to be substituted with values of 3, 4, and 5, respectively Table 1.

**Table 1:** Definitions of MAS for grading muscle spasticity.

**Grade**	**Modified ashworth scale (28)**	**Values**
0	No increase in muscle tone;	0
1	Slight increase in muscle tone, manifested by a catch and release or by minimal resistance at the end of the range of motion when the affected part is moved in flexion or extension;	1
1+	Slight increase in muscle tone, manifested by a catch, followed by minimal resistance throughout the remaining (less than half) range of motion;	2
2	More marked increase in muscle tone through most of the range of motion, but affected part easily moved;	3
3	Considerable increase in muscle tone, passive movement difficult;	4
4	Affected part(s) rigid in flexion or extension.	5

*For convenience of statistical analysis, a Modified Ashworth Scale grade 1+ is to be substituted with a value of 2, and grade 2, 3 and 4 are matched to 3, 4, and 5, respectively*.

(1) Recovery: Muscle tension had completely returned to normal;
(2) Obvious effect: The muscle tension had not returned to normal, but muscle tension had decreased by two levels;
(3) Effective: Muscle tension had decreased by 1 level;
(4) Inefficacy: No change in muscle tone pre- and posttreatment.

#### Secondary Outcome Measures

Electromyography (EMG), surface electromyography (sEMG), MyotonPRO digital muscle function evaluation system, and infrared thermal imaging (IRT) are to be used for the evaluation.

1. **H reflex:** H reflex of the median nerve is to be detected using the Schwarzer topas EMG system (Natus Medical Incorporated, Pleasanton, CA, USA). The recording must be conducted from the flexor carpi radialis as follows: the recording electrode is to be placed at the mid-upper 1/3 junction of the medial humeral epicondyle and the radial styloid process line, the reference electrode is to be placed at the tendon, the ground electrode is to be placed at the olecranon, and the stimulation electrode is to be placed at the median nerve proximally to the elbow. The maximal amplitude of H-reflex (H-max) and M-response (M-max) are to be recorded. The H-max/M-max value, which can reflect the excitability change of the α-motor neuron, is to be calculated to detect changes in response to the effect of rESWT on the spasmodic muscle.
2. **Motor Nerve Conduction Velocity:** The same apparatus is to be used, but the electrodes are to be placed differently. The recording electrodes must be placed on the abductor pollicis brevis muscle belly, and the stimulating electrode must be placed on the median nerve at wrist and elbow level to record distal motor latency, MCV, and amplitude. This assessment is aimed at determining whether rESWT causes nerve injury and evaluating its safety.
3. **Root-Mean-Square Value:** An sEMG recording is to be obtained using bipolar Ag/AgCl surface electrodes (MyoMove, Northam Electric Co., Ltd., Shanghai, China) for the sEMG assessment. The patient is to be treated while in a sitting position. The electrodes are to be placed on the fullest part of the upper limbs muscle belly. The locations at which the stimulating and recording electrodes are to be placed must be rubbed with 70% alcohol to reduce skin impedance. Electrode placement is to be done as recommended by Surface EMG Non-invasive Muscle Assessment and the International Society of Electrophysiology and Kinesiology. With the upper limbs of a patient comfortably placed on the treatment table, the resting sEMG activity of the musculus biceps brachii (BM) and flexor carpi radialis are to be measured under stable and static conditions for 30 s at a time. Also, the RMS of biceps brachii and flexor carpi radialis is calculated. RMS can be used to objectively and quantitatively evaluate the condition of muscle spasms, with a higher value indicating a more severe muscle spasm.
4. **Integrated Electromyogram and Co-contraction Ratio:** The patient is seated with the upper body fixed to the seat with a wide nylon strap. The elbow of the patient is at a 45° flexed position and wrist in a neutral position, and the hand holds the handle of the arm strength test joystick of Biodex System 3® dynamometer (Biodex Medical Systems, Shirley, NY, USA). The electrodes are to be placed on the muscle belly of BM and musculus triceps brachii (TM). Training is to be performed for 1 min before the assessment to help patients familiarize themselves with the process. During the test, the patients are to be asked to stretch the elbow joint with maximum strength for 10 s to measure maximum isometric voluntary contraction (MIVC) of TM (44). The iEMG and CR of BM and TM are to be recorded for 20 and 5 s before and after contraction, respectively, as the basic control. The test is to be carried out three times, with an interval of 5 min between each application, and the maximum value is to be recorded. The assessment includes the iEMG of the BM and TM when the elbow is stretched for MIVC, and then the CR is calculated. The iEMG value can reflect the total amount of muscle discharge per unit time and is mainly used to analyze the contraction characteristics of muscles per unit time. CR refers to the proportion of antagonist muscles during the process of active muscle contraction (34), although it is well known that increased synergistic contraction of antagonistic muscles is a common phenomenon in stroke patients. The decrease of CR within MIVC of the elbow indicates a reduction in the strength of the biceps tendon.
  $$\begin{array}{l} \text{CR}=\frac{\text{iEMG of BM }}{\text{iEMG of BM }+\text{ iEMG of TM}}\times 100\% \end{array}$$
5. **MyotonPRO Digital Muscle Function Assessment System:** The MyotonPRO system (Muomeetria Ltd., Tallinn, Estonia, EU) performs a noninvasive measurement of the functional state of skeletal muscles and biological soft tissues, which can quantitatively assess the functional status of muscle tension and elasticity (45, 46). The patient is asked to completely relax while lying in the supine position on a mat, with the forearm in the mid position and the elbow joint extended (if the muscles cannot be fully extended, the forearm is supported on both sides to maintain the forearm neutral position). The measurement point of the BM is tested perpendicular to the skin surface. Measurement is taken once every 1 min, for three times in total, and the average is obtained. The parameters required are (47): F—natural damped oscillation frequency (hertz) to describe muscle tension; C—Creep is the ratio of muscle relaxation time to deformation time, also called Deborah number; R—Muscle internal mechanical pressure release time (millisecond); and C and R reaction to the viscoelasticity of the muscle.
6. **Infrared Thermal Imaging:** A non-invasive and non-contact infrared thermal imaging system (Baotonghua Medical Devices Co., Ltd. Chongqing, China) is to be used to detect changes in local trophic conditions related to blood microcirculation and surface temperature distribution. The instrument has a thermal sensitivity of 0.1 °C. Before measurement, all clothes covering the area to be examined must be removed, and the patients are allowed to adapt to the room for 15–20 min. The room is to be kept quiet with a temperature ranging from 22 to 27 °C and relative humidity below 45%. Then, a thermovision camera is positioned perpendicular to the area of the biceps surface on the anterior side of the arm at a distance of 1 m. The IRT value is obtained in degrees Celsius. The lower the surface temperature, the worse local blood circulation and trophic conditions of muscles.

### Participants

Patients with flexor spasticity of the upper limb after stroke (Table 2).

**Table 2:** Flow chart of the study.

**Items/Time points**	**Enrollment**	**Allocation**	**Post-allocation**		
	***-t1***	**Baseline**	***t1***	***t2***	***t3***
Basic medical history	×				
Eligibility screen	×				
Informed consent	×				
Allocation(Group A/Group B)		×			
Intervention(Group A/Group B)		×			
Assessments	MAS		×	×	×	×
	H reflex		×			×
	MCV		×			×
	RMS		×	×	×	×
	iEMG		×	×	×	×
	CR		×	×	×	×
	MyotonPRO		×	×	×	×
	IRT		×	×	×	×

*Group A, active rESWT; Group B, sham-placebo rESWT; MAS, Modified Ashworth Scale; MCV, Motor nerve conduction velocity; RMS, Root mean square value; iEMG, Integrated electromyogram; CR, co-contraction ratio; IRT, Infrared Thermal Imaging; t1/ t2/ t3, at 24 hours after the first/ second/ third radial extracorporeal shock wave therapy*.

### Recruitment

Direct recruitment from Zhujiang Hospital.

### Sample Size Calculation

The known effectiveness of poststroke spasticity treatment using conventional methods is 64.5%, whereas preliminary experiments with ESWT have shown expected effectiveness of 80%, with two-tailed tests of a significance level of α = 0.05 and β = 0.1, which is expected to fall off at a rate of 10%, using professional software Advisor nQuery sample size estimation, for a sample size of 45 cases in each group, which can be considered as a loss rate of approximately 10%. Therefore, for this study, a sample size of 50 cases in each group was decided on, with a total sample size of 100 cases.

### Randomization

Eligible patients are to be numbered sequentially based on enrollment sequence and randomly assigned to group A (active rESWT) or group B (sham-placebo rESWT), with a total of 50 patients in each. Random numbers are to be generated using a computer software program run by an external statistician. Each of the random numbers and the group assignment are to be written on a piece of paper and enclosed in a sealed envelope.

### Blinding

This study uses a double-blinded design, in which interventions are performed by one physiotherapist and patients are evaluated by another physician who is unaware of the treatment or grouping. Neither of them will participate in the subsequent data analysis.

After the statistical judgment is completed, unblinding will be performed to expose the experimental group and the control group. In general, emergency unblinding is not considered.

### Data Collection Methods

The evaluation index is to be collected before and after treatment is performed on all patients, with raw data recorded on case report forms (CRFs) in a timely, complete, accurate, and clear manner. To improve subject compliance, subjects who can complete the entire procedure in accordance with the protocol are to be provided with additional rehabilitation assessments and rehabilitation recommendations. If a participant chooses to withdraw, they will be asked to provide reasons, and the reasons for withdrawal are to be recorded.

### Data Management

An EPIDATA 3.2 database will be used to manage data, for which input and proofreading will be performed by two researchers independently, leading to double data entry and storage.

### Statistical Methods

The primary comparisons for MAS will be made using repeated measures mixed-effect model with terms of treatment, time, and corresponding baseline values as covariates. We will first examine the intervention by time interaction and then proceed to the main effects model with only group and time.

Repeated analysis of variance with *post-hoc* test will be used to compare changes between active rESWT intervention and sham rESWT intervention groups from baseline to the end of follow-up when data are normally distributed, and the Mann–Whitney U-test will be used when data are not normally distributed. A chi-square test will be used for dichotomous variables.

In secondary analyses, repeated measures mixed model will also be used to examine the associations between treatments and repeated measures outcomes. Additionally, linear regression and/or logistic regression analyses will be used to assess the associations between treatments and changes or increases in outcomes from baseline to the end of follow-up in univariate and multivariate modeling adjusted for relevant covariates.

All data will be analyzed using intention-to-treat principles. Multiple imputation by chained equations will be used to address missing data caused by loss to follow-up and non-responses. Per protocol analyses will also be performed in the participants who complete other assessments, including Hmax/Mmax, MCV, RMS, iEMG, CR, MyotonPRO, and IRT. Statistical analysis will be performed using SPSS software (version 20.0) and the significance level set at *p* < 0.05.

### Data and Safety Monitoring

Original CRFs will be archived and stored with corresponding subject codes after the completion of data entry and review. A Data Monitoring Committee (DMC), composed of clinicians and biostatisticians, without any competing interests, will monitor the safety and progress of the trial.

### Harm and Audit

The researchers are obliged to take necessary measures to protect the safety of the subjects. The subjects are informed that there may be adverse events (AEs) such as pain and hematomas during and after ESWT through oral notification and informed consent form. If an AE occurs during the trial, the investigator should take appropriate measures, record it in the CRF, and explain whether it has a correlation with the intervention. The incidence of AEs between groups A and B should be compared after the trial. If serious adverse events occur during the clinical trials, the investigator should immediately take appropriate treatment measures and report to the sponsor, the Ethics Committee (EC), and DMC in a timely manner.

During the trial, Tao Fan will be responsible for communicating with relevant parties (such as other investigators, trial participants, journals, and regulatory authorities) if there is a need for subsequent modification of important experimental protocols, and any modification of the trial protocol should be approved by the EC. The EC and DMC will periodically review the experimental behavior to safeguard the rights of the subjects involved in the clinical trial, to ensure the accuracy and completeness of the test records and reported data, and to ensure consistency with the protocol approved. If serious adverse events caused by interventions occur during the trial, the EC has the right to propose a modification of the trial protocol or even terminate the trial.

### Trial Status

This is the second version of the study protocol dated October 20, 2017. This trial was registered on May 14, 2018. The first patient was recruited on October 18, 2018. At the time of manuscript submission, a total of 50 patients have been recruited, and we hope to complete recruitment within 3 years. After patient recruitment is completed, all data will be statistically analyzed, and a research article will be written and submitted.

### Additional File

**Additional file 1:** Standard Protocol Items: Recommendations for Interventional Trials 2013 Checklist: recommended items to be addressed in a clinical trial protocol and related documents.^*^

## Discussion

Stroke survivors with spasticity suffer substantial mental, physical, and financial stress. Effective spasticity treatment will likely increase their functioning and their health-related quality of life. Clinical studies (48–50) have shown that ESWT may improve the muscle spasm of stroke patients without serious adverse reactions. A meta-analysis (51) found that adding the ESWT to conventional therapy provides an additional benefit for reducing upper limb spasticity, and the results may be optimal given the ESWT at the subacute phase, but the mechanism remains unclear. Many studies conducted in recent years have been investigating the biological effects of ESWT (52–55). In the past, the mechanism by which ESWT acts on musculoskeletal diseases was assumed to be mechanical decomposition, just like extracorporeal shock wave lithotripsy. However, further clinical observations and experimental results have suggested that ESWT can promote neovascularization, the release of growth factors, the differentiation of mesenchymal stem cells, and the production of endogenous nitric oxides (15, 56, 57), which can decrease the intrinsic stiffness of connective tissue, increase muscle elongation, improve tissue microcirculation, and change the formation of neuromuscular junctions of the peripheral nervous system (55, 58, 59), to achieve encouraging clinical results.

We know that poststroke spasticity is related to the hyperexcitability of stretch reflexes and the changes in muscle properties (60). There are a few hypotheses that attempt to explain the effect of ESWT reducing spasticity. Daliri et al. (48) reported a significant improvement of the wrist flexor muscles spasticity assessed by the α-motor neuron excitability and the H-reflex (H-max/M-max value) of post-ESWT after stroke. However, some studies (61, 62) found that the effect of ESWT did not relate to α-motor neuron excitability. The hypothesis on spinal cord excitability seems to be weak, which adds some support to the hypothesis that ESWT affects periphery biomechanical properties of the hypertonic muscles. Lee et al. (63) assessed the effect of a single session of ESWT on patients with stroke by an ultrasonographic assessment on gastrocnemius before the treatment and 30 min and 1 and 4 weeks after treatment, and they found that the mean scores of Achilles tendon length, muscle thickness, and pennation angle were decreased, whereas muscle fascicle length increased in the ESWT group at any follow-up time. The study showed that muscular architecture parameters related to muscle fiber mechanics were improved after ESWT. Also, this may be due to the mechanical vibration of ESWT reducing intrinsic stiffness of connective tissue and promoting the local release of angiogenetic factors and growth factors, resulting in the addition of sarcomere and increased muscle length. Furthermore, ESWT can induce nitric oxides, which seem to play an important role in spasticity relieving mechanisms through involving in the formation of neuromuscular junction formation in the peripheral nervous system and in physiological functions of the central nervous system (15, 59). A new study by Leng et al. (64) applied passive torque measurement combined with biomechanical modeling, myotonometer measurements, and electrical impedance myography to assess the changes of muscle properties induced by ESW in the spastic wrist joint, then concluded that both the neural and peripheral components played a role in muscle spasticity, and ESWT may be more effective in addressing the peripheral component of spasticity muscle.

Based on the propagation pattern and device of the waves, ESWT can be classified as fESWT and rESWT. There are some common mechanisms of biological action for fESWT and rESWT, but they also differ in penetration depth and other physical properties related to clinical effect. rESWT lacks the characteristic features of shock waves, such as short rise-time, high peak pressure, and non-linearity (65). In addition, rESWT had a more superficial effect compared with focused shock waves that penetrated and can focus their energies much deeper into the tissue (16). Compared with fESWT, which can penetrate and focus its energies more rapid and much deeper into the tissue (pressure increasing under 10 ns, reaching 100–1,000 bars with absorption to 12 cm), the pressure of rESWT increases slowly and has a more superficial effect (pressure increasing up to 5 μs, reaching 1–10 bars and absorbed to 3 cm). Wu et al. (30) compared the effect of fESWT and rESWT for the treatment of spastic equinus in patients with stroke; they concluded that the effect of rESWT was superior to the fESWT in improving the ankle passive range of motion and plantar contact area during gait, whereas both of them showed similar improvement in the spasticity of the gastrocnemius muscle; there was no significant difference in changes for MAS scores between the two groups. Other significant differences of rESWT and fESWT in the clinical application effects are still being observed.

Through the use of new assessment techniques, this trial is designed to generate a considerable amount of outcome data to clarify its effects on the neural and peripheral contribution of muscle spasticity and provide strong supporting evidence for the effectiveness of rESWT for the management of spasticity after stroke. We hypothesize that after active rESWT, patients will show greater improvement in upper limb muscles compared with patients who have received sham-placebo rESWT treatment. rESWT would be an attractive alternative to traditional methods, and the results could provide guidance and support for the further study of potential mechanisms.

Our study also has some limitations. First, this study is a single-center clinical study, which was slow to collect subjects. Second, the rESWT interference only three sessions due to limited length of hospital stay, which may affect clinical curative during the treatment phase. Third, the follow-up time of this study is short due to the short hospital stay of the patients.

## Ethics Statement

The studies involving human participants were reviewed and approved by the Institutional Ethics Committee of the Zhujiang Hospital of Southern Medical University has approved our study (Reference Number: 2017-KFYXK-003). The patients/participants provided their written informed consent to participate in this study.

## Author Contributions

All authors contributed to the design of the study protocol. TF is responsible for this study. GH conceived and developed the study design. XYZ and RC drafted the trial protocol and prepared the manuscript. PCH, PZ, and MYW revised the protocol. XJZ, RDL, RHL, and XZ are responsible for data acquisition and analyses. All authors have read and approved the final manuscript.

## Funding

Guangdong Provincial Science and Technology Department self-financing fund science and technology plan project (2017ZC0121). This research project has been peer-reviewed by the Guangdong Provincial Science and Technology Department and has been approved for set up, but there is no fund sponsorship from it for this project.

## Conflict of Interest

The authors declare that the research was conducted in the absence of any commercial or financial relationships that could be construed as a potential conflict of interest.

## Publisher's Note

All claims expressed in this article are solely those of the authors and do not necessarily represent those of their affiliated organizations, or those of the publisher, the editors and the reviewers. Any product that may be evaluated in this article, or claim that may be made by its manufacturer, is not guaranteed or endorsed by the publisher.

## Abbreviations

AE: adverse event
BM: musculus biceps brachii
BTX: botulinum toxin
CR: co-contraction ratio
CRFs: Case report forms
DMC: Data Monitoring Committee
EC: Ethics Committee
EMG: Electromyography
ESWT: Extracorporeal shock wave therapy
fESWT: focused extracorporeal shock wave therapy
H-max: the maximal amplitude of H-reflex
iEMG: Integrated electromyogram
IRT: infrared thermal imaging
MAS: Modified Ashworth Scale
MCV: motor nerve conduction velocity
MIVC: maximum isometric voluntary contraction
M-max: the maximal amplitude of M-response
rESWT: radial extracorporeal shock wave therapy
RMS: root mean square
sEMG: surface electromyography
TM: musculus triceps brachii.
